# Supplementary material for: The interactome of intact mitochondria by cross-linking mass spectrometry provides evidence for coexisting respiratory supercomplexes
Source: Mol Cell Proteomics. 2017 Dec 8;17(2):216–32. doi: 10.1074/mcp.RA117.000470 (PMC5795388; doi:10.1074/mcp.RA117.000470)
Supplement: Supplemental Data [file supp_RA117.000470_133922_0_supp_23978_fzffwf.zip › spectra_annotation/mito_spectra_annotation/345-1-9-1-13-1.pdf]

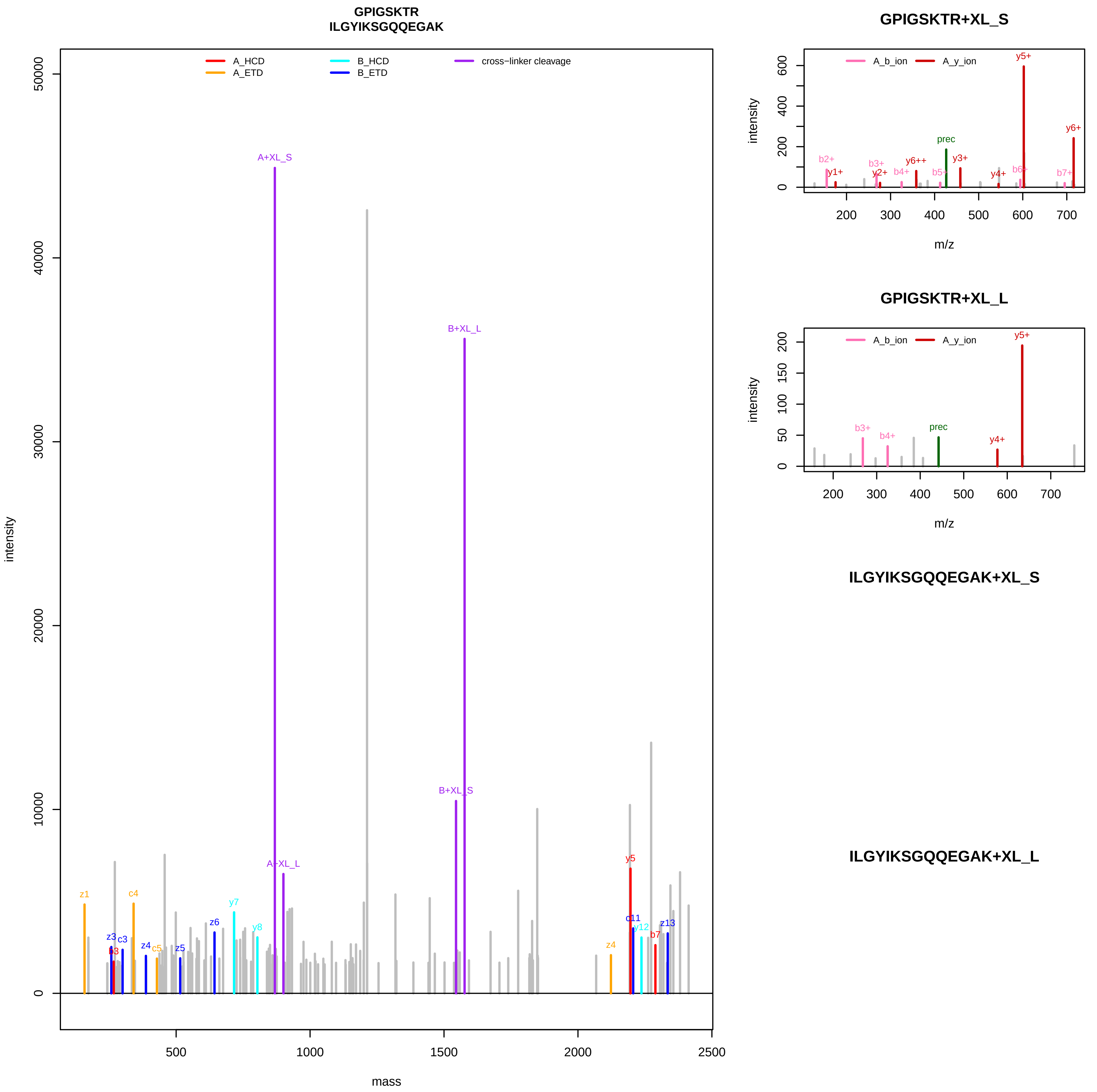

GPIGSKTR
ILGYIKSGQQEGAK
A_HCD
A_ETD
B_HCD
B_ETD
cross−linker cleavage
A+XL_S
B+XL_L
B+XL_S
A+XL_L
z1
c4
z3
c3
b3
z4
c5
z5
z6
y7
y8
y5
c11
y12
b7
z13
intensity
mass
0
10000
20000
30000
40000
50000
500
1000
1500
2000
2500
GPIGSKTR+XL_S
A_b_ion
A_y_ion
y5+
y6+
prec
y3+
b2+
y1+
b3+
y2+
y6++
b4+
b5+
y4+
b6+
b7+
intensity
m/z
GPIGSKTR+XL_L
A_b_ion
A_y_ion
y5+
b3+
b4+
prec
y4+
intensity
m/z
ILGYIKSGQQEGAK+XL_S
ILGYIKSGQQEGAK+XL_L
